# Repair of Acute Patellar Tendon Rupture Using an Internal Brace Technique

**DOI:** 10.1155/2021/1086625

**Published:** 2021-11-18

**Authors:** Aki Fukuda, Shigeto Nakazora, Akinobu Nishimura, Ko Kato

**Affiliations:** ^1^Department of Orthopaedic Surgery, Suzuka Kaisei Hospital, 112-1 Kou, Suzuka, Mie 513-0836, Japan; ^2^Department of Orthopaedic and Sports Medicine, Mie University Graduate School of Medicine, 2-174 Edobashi, Tsu, Mie 514-8507, Japan

## Abstract

Acute patellar tendon rupture is a serious injury, resulting in the disruption of the knee extensor mechanism. Many authors recommend augmented repairs of patellar tendon ruptures to allow early active rehabilitation. An internal brace technique, which is a ligament augmentation using high-strength suture tape and knotless anchors, has been used as augmentation for the primary tendon or ligament injury. A case of acute patellar tendon rupture in a Judo player, who was successfully treated with primary repair and augmentation using an internal brace technique, is presented. In this case, the patient regained full function of the knee and returned to full sports activities postoperatively. An internal brace technique provides biomechanical stability of the repaired tendon without donor site morbidity and could be an effective procedure for the treatment of acute patellar tendon rupture.

## 1. Introduction

Acute patellar tendon rupture is a relatively uncommon injury that is generally associated with tendinopathy caused by repetitive microtrauma, prexisting systemic disease, or steroid use [1]. Several surgical procedures for acute patellar tendon rupture, including primary repair, augmented repair, or primary reconstruction, have been proposed [2, 3]. Many authors recommend augmented repairs of patellar tendon ruptures to allow early active rehabilitation [4–9], but the proper surgical procedure for patellar tendon rupture remains controversial. Recent studies have shown excellent clinical outcomes after primary repair with suture tape augmentation for knee, ankle, and elbow ligament injuries [10–12]. An internal brace technique is a ligament augmentation using high-strength suture tape and knotless anchors, which provides biomechanical stability of the repaired tendon without donor site morbidity. In addition, this technique facilitates immediate postoperative mobilization and early functional recovery. A case of acute patellar tendon rupture in a Judo player, who was successfully treated with augmented repair using an internal brace technique, is presented.

## 2. Case Presentation

**Figure 1:**
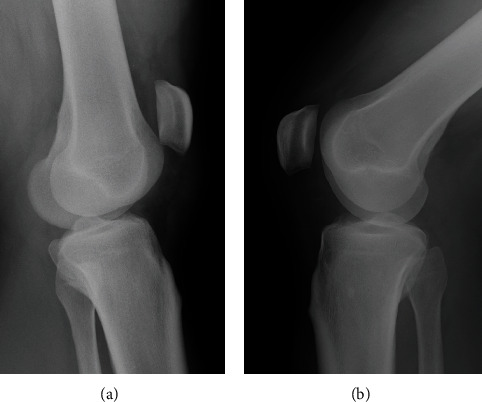
Preoperative lateral radiographs of the (a) left knee and (b) right knee.

**Figure 2:**
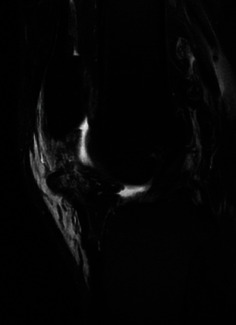
Fat-suppressed T2-weighted sagittal magnetic resonance image of the left knee shows rupture of the left patellar tendon.

**Figure 3:**
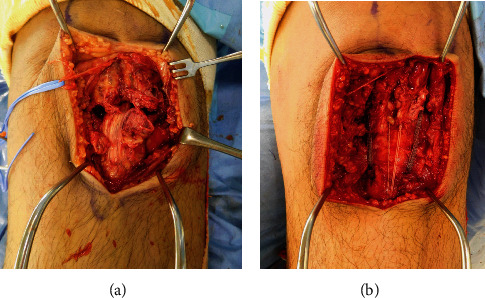
Intraoperative view of the left knee (a) and final view of the augmented repair using an internal brace technique (b).

A 25-year-old man (weight: 126 kg, height: 185 cm, BMI: 36.8 kg/m^2^) felt a popping sensation and severe pain in the left knee when he twisted his left leg with the knee flexed during Judo activity. He had no history of chronic disease or steroid use. He could not actively extend his left knee. Physical examination of the left knee showed swelling, a high patella, and a palpable gap below the patella. Plain radiographs of the left knee showed patella alta (Figure 1). Magnetic resonance imaging (MRI) confirmed a complete rupture of the proximal patellar tendon (Figure 2). Surgery was performed in the supine position under spinal anesthesia. A straight anterior incision was made from the inferior pole of the patella to the tibial tuberosity. The patellar tendon and extensor retinaculum were completely torn at the proximal portion (Figure 3(a)). Two 5.5 mm corkscrew suture anchors with two no. 2 FiberWires (Arthrex, Naples, FL) were placed just lateral to the center of the inferior pole of the patella. All suture limbs were sutured into the patellar tendon using the Krackow technique and were fixed to the tibial tubercle using one 2.9 mm Pushlock anchor (Arthrex). Finally, an internal brace technique was used to augment and protect the repaired tendon. Two 4.75 mm SwiveLock suture anchors loaded with a 2 mm suture tape (Arthrex) were placed just lateral and medial to the patellar tendon at the inferior portion of the patella. The free ends of the suture tape were passed through a second SwiveLock suture anchor, and the suture anchor was fully inserted just lateral and medial to the tibial tubercle with the knee at 60° of flexion (Figure 3(b)). Correct placement of the patella was confirmed under fluoroscopic control, as was the stability of the repaired tendon through the range of motion.

**Figure 4:**
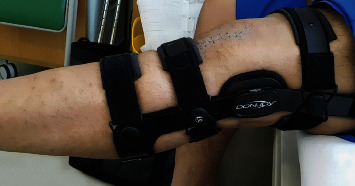
The patient has regained full active extension at 1 month postoperatively.

**Figure 5:**
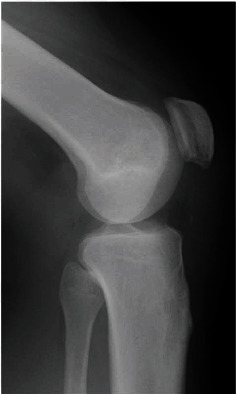
Postoperative lateral radiograph of the left knee at follow-up 12 months after the operation.

For postoperative rehabilitation, the patient was placed in a locked hinged knee brace in full extension and was also instructed to perform isometric muscle training immediately after surgery. Range of motion exercise was immediately started between 0° and 90° for 2 weeks and then from 0° to 120° for 6 weeks. Partial weight-bearing was started 3 weeks after surgery, and full weight-bearing was allowed after 6 weeks. At 1 month postoperatively, full active extension could be achieved without an extension lag (Figure 4). At 2 months postoperatively, the patient regained full function of the knee and returned to work. Jogging was encouraged after 3 months, and return to full sports activities was allowed at 5 months postoperatively. At the final follow-up, 12 months postoperatively, he was symptom-free with a full range of motion, and he regained preinjury functional levels (Figure 5). No complications were reported during the postoperative period.

## 3. Discussion

Acute patellar tendon rupture is a serious injury, resulting in the disruption of the knee extensor mechanism. Unlike a quadriceps tendon tear, patellar tendon rupture commonly occurs in active subjects less than 40 years of age. A previous study showed that the force required to disrupt a patellar tendon is 17.5 times the body weight [13]. In the present case, the patient was a healthy young athlete with a high BMI, and the mechanism of the injury might have been a violent eccentric contraction of the quadriceps muscle with the knee flexed during Judo activity.

The treatment goal for acute patellar tendon rupture is restoration of the extensor mechanism of the knee. Recent studies have shown excellent clinical outcomes for acute patellar tendon rupture after augmented repair. In a biomechanical study, augmented repair yielded significantly less gap formation and higher ultimate failure loads compared with simple repair [14]. However, an augmented repair technique has several disadvantages. Augmentation using an autograft has donor site morbidity. Furthermore, the placement of rigid devices such as metal wire or synthetic material usually requires a second surgery for removal due to the soft tissue irritation or hardware breakage. An augmentation device was usually passed through the patellar bone tunnel or the quadriceps tendon in a figure-of-eight fashion. Augmentation from the proximal patella to the tibial tubercle changes the moment arm and increases patellofemoral pressure, which may result in postoperative stiffness or patellofemoral complications.

A recent report presented the surgical technique using an internal brace technique for the treatment of patellar tendon rupture [15]. An internal brace technique is a ligament augmentation using high-strength suture tape and knotless anchors, which provides biomechanical stability of the repaired tendon without donor site morbidity. In addition, an internal brace technique requires the minimum length of augmentation from the inferior portion of the patella to the tibial tubercle, which maintains normal anatomical structure and appropriate patellofemoral tracking. Concerning the surgical technique, overtightening should be avoided to obtain a better clinical outcome. Previous reports have shown that an augmentation device was fixed at a different angle of knee flexion, ranging from 0° to 90°. Lindy et al. reported cases of postoperative patellofemoral chondrosis after augmentation using Mersilene tape that was tied with the knee in full extension [16]. Therefore, it might be reasonable that the augmentation device should be fixed at some degrees of knee flexion, but the appropriate fixation angle of the knee remains controversial. Clinically, Marder and Timmerman recommended that all repairs be performed to allow knee flexion to 60° to prevent overtightening of the patellar tendon [2]. Several biomechanical studies have shown that the patellar moment arm length is increased in the range from 30° to 60° [17]. Moreover, a study of in vivo patellar tendon kinematics during weight-bearing showed that the relative elongation of the patellar tendon increased up to 60° and then decreased as the knee flexed up to 120° [18]. In the present case, the augmentation using the suture tape was fixed with the knee in 60° of flexion, and there were no postoperative complications, such as stiffness, patellofemoral pain, or rerupture.

## 4. Conclusion

A case of acute patellar tendon rupture in a Judo player who was successfully treated with primary repair and augmentation using an internal brace technique was presented. An internal brace technique provides biomechanical stability of the repaired tendon without donor site morbidity and allows early postoperative rehabilitation and early functional recovery. This technique could be a safe and effective procedure for the treatment of acute patellar tendon rupture.

## Data Availability

The data used to support the findings of this study are included within the article.
